# Disparities in access to COVID-19 vaccine in Verona, Italy: a cohort study using local health immunization data

**DOI:** 10.3389/fpubh.2023.1167414

**Published:** 2023-06-15

**Authors:** Roberto Benoni, Anna Sartorello, Francesca Moretti, Francesco Marchiori, Luciana Accordini, Chiara Postiglione, Viviana Coffele, Stefano Tardivo

**Affiliations:** ^1^Department of Diagnostics and Public Health, University of Verona, Verona, Italy; ^2^Department of Prevention, Unità Locale Socio-Sanitaria (ULSS) 9, Verona, Italy

**Keywords:** COVID-19, health inequities, migrants, SARS-CoV-2, healthcare access, COVID-19 vaccine, claims database

## Abstract

**Introduction:**

Migrant populations worldwide were disproportionately impacted by the COVID-19 pandemic. Although substantial resources have been invested in scaling COVID-19 vaccination campaigns, globally vaccine rate and uptake remained low among migrants from across many countries. This study aimed to explore the country of birth as a factor influencing access to the COVID-19 vaccine.

**Methods:**

This retrospective cohort study included adults vaccinated against SARS-CoV-2 receiving at least one dose in the Verona province between 27 December 2020 and 31 December 2021. Time-to-vaccination was estimated as the difference between the actual date of each person's first dose of COVID-19 vaccination and the date in which the local health authorities opened vaccination reservations for the corresponding age group. The birth country was classified based on both the World Health Organization regions and the World Bank country-level economic classification. Results were reported as the average marginal effect (AME) with corresponding 0.95 confidence intervals (CI).

**Results:**

During the study period, 7,54,004 first doses were administered and 5,06,734 (F = 2,46,399, 48.6%) were included after applying the exclusion criteria, with a mean age of 51.2 years (SD 19.4). Migrants were 85,989 (17.0%, F = 40,277, 46.8%), with a mean age of 42.4 years (SD 13.3). The mean time-to-vaccination for the whole sample was 46.9 days (SD 45.9), 41.8 days (SD 43.5) in the Italian population, and 71.6 days (SD 49.1) in the migrant one (p < 0.001). The AME of the time-to-vaccination compared to the Italian population was higher by 27.6 [0.95 CI 25.4–29.8], 24.5 [0.95 CI 24.0–24.9], 30.5 [0.95 CI 30.1–31.0] and 7.3 [0.95 CI 6.2–8.3] days for migrants from low-, low-middle-, upper-middle- and high-income countries, respectively. Considering the WHO region, the AME of the time-to-vaccination compared to the Italian group was higher by 31.5 [0.95 CI 30.6–32.5], 31.1 [0.95 CI 30.6–31.5], and 29.2 [0.95 CI 28.5–29.9] days for migrants from African, European, and East-Mediterranean regions, respectively. Overall, time-to-vaccination decreased with increasing age (p < 0.001). Although both migrants and Italians mainly used hub centers (>90%), migrants also used pharmacies and local health units as alternative sites (2.9% and 1.5%, respectively), while Italians (3.3%) and migrants from the European region (4.2%) relied more on family doctors.

**Conclusion:**

The birth country of migrants influenced access to COVID-19 vaccine both in terms of time-to-vaccination and vaccination points used, especially for the LIC migrant group. Public health authorities should take socio-cultural and economic factors into consideration for tailored communication to people from migrant communities and for planning a mass vaccination campaign.

## 1. Introduction

Data from the past 2 years have shown that asylum seekers and migrants in high- and upper-middle income countries were disproportionately affected by the coronavirus disease 2019 (COVID-19) pandemic; they generally experienced a higher risk of infections and had worse outcomes (1, 2). In European countries, 30% of migrants live in poverty, and among the general population, socio-economic status and ethnicity were independently associated with an increased risk of COVID-19 morbidity and mortality (3, 4). In particular, Black and Asian ethnic groups experienced a higher risk of infection than other groups (5).

In the northern Italian region of Veneto, the infection rate from severe acute respiratory syndrome coronavirus 2 (SARS-CoV-2) was significantly higher among migrants from Central and South America and Central and South Asia; hospitalization rate was also higher for all migrant populations compared to Italians, the only exception being migrants from high-income countries (HIC) (6).

In December 2020, the European Center for Disease Prevention and Control (ECDC) included migrants and refugees as potential target populations for COVID-19 vaccination campaigns (7). The Council of Europe has urged that access to vaccination should be adapted to the needs of individuals in vulnerable situations. The council suggested including low-income migrant workers and individuals without a permanent place of residence or with insecure legal status in target groups (8).

Italy rolled out its vaccination campaign on 27 December 2020. At first, vaccinations were offered to health workers, the older adults (over 65 years), and individuals with other risk factors, i.e., comorbidities. Vaccination was then offered to the entire population (9). Italy's strategic plan for this campaign neither explicitly mentioned nor included migrants under any of its targeted priority groups. The right to vaccination regardless of the juridical status is stated by the Italian Drug Agency (AIFA), in line with the Italian Constitution which defines health as a fundamental right for the individual (10–12).

It is important to note that vaccinations should be made available to all, especially for disadvantaged groups who may be more vulnerable to diseases and to complications related to both communicable and non-communicable conditions. Vaccinations should be made not only available but also accessible for these at-risk populations (13).

Geographic origin is a well-known determinant of under-vaccination among migrants. Studies on these populations usually address vaccination rate, barriers to vaccination, or vaccination interventions. However, the complex issue of access to healthcare involves not only accessibility to these services but also acceptability, affordability, and reception (14). The need to investigate the relative contributions of these different factors to suboptimal vaccine uptake in migrant populations has been highlighted, with the aim of guiding the development of evidence-based interventions to improve vaccine equity (15). The primary objective of this study was to explore differences in access to the first dose of the COVID-19 vaccine, during the first year of the COVID-19 vaccination campaign in Italy (2021), looking at factors such as the time to vaccination and the influence that the birth country may have had on vaccine uptake. A secondary objective was to assess the association between birth country and vaccination points (the place where the COVID-19 vaccine was given). For this second objective, the study explored if the type of structure (hub centers, pharmacies, local health units, etc.) and the distance from an individual's place of residence to a vaccination point mattered in the receipt of the vaccine.

## 2. Materials and methods

### 2.1. Study design

A retrospective cohort study was conducted to explore the potential impact that the birth country may have on access to the COVID-19 vaccine.

### 2.2. Ethical approval

The inclusion in the study analysis did not require any additional exams in addition to those normally performed for routine clinical practice. The research was performed following the ethical standards of the 2000 Declaration of Helsinki and the Ethical Committee of AULSS 9 Scaligera as approved by the European Union's Good Clinical Practice Standards, Verona, on 12 September 2022 (protocol number 54523).

### 2.3. Setting

The Verona province includes 98 municipalities and has an estimated population of 9,27,108 people (16). A single local health trust, comprising four health districts, administers disease prevention plans and healthcare to the general population of the Verona province.

Migrants in the Verona province numbered 1,11,030 as of 1 January 2022; this represented 12.0% of the population at that time. The largest migrant group came from Romania, accounting for 29.7% of all migrants, This was followed by Morocco (12.0%), Sri Lanka (formerly Ceylon, 9.2%), and Albania (5.7%) (16).

In Italy, the COVID-19 vaccination campaign was opened at different times according to age and comorbidities, based on defined risk categories (17). The category with age as the only risk factor was divided by the local health authorities into six groups, for which the opening dates to request and receive the first dose of the vaccine were as follows (18):

- 15 February 2021, over 80 years-old
- 15 April 2021, 60–79 years-old
- 29 April 2021, 40–59 years-old
- 07 June 2021, 12–39 years-old
- 16 December 2021, 5–11 years-old.

The vaccination strategy adopted at the regional level was based on large vaccination centers, initially opened for mass vaccination of the population. Subsequently, vaccination points were established at the primary health care level, such as pharmacies and local health units (LHUs).

### 2.4. Data source and collection

The Verona Local Health Vaccination Database stores data from all vaccinations administered by every vaccination point in the province of Verona. During the study period, all COVID-19 vaccinations were offered and administered free of charge. All vaccination points operated and used the same software Regional Vaccination Information System (SIAVr) to record their vaccination data. From this local database, data were then sent to the regional database Veneto Region Vaccination Database (19).

SIAVr collected the following information for each individual vaccinated in the Verona province between 27 December 2020 and 31 December 2021: age, sex, municipality of residency, place of birth, date and place of vaccination, number of COVID-19 vaccinations received, risk categories, previous COVID-19 infections, and second dose exemption, if consistent. For the study analyses, data were de-identified to preserve anonymity and then retrieved.

### 2.5. Population

Individuals older than 18 years of age, who had received the first dose of the COVID-19 vaccine at any of the vaccination points in the province of Verona in the study period, and were registered in the regional information system, were included in the study analysis.

To reduce bias in the estimation of the time-to-vaccination variable, the following cases were excluded based on the information available in the local health immunization database:

- Individuals with previous COVID-19 infection since they could wait up to 12 months to receive the vaccine
- Individuals with risk factors due to comorbidities or vulnerable health status because they were vaccinated earlier than their age group (i.e., diabetics and cancer patients) (20)
- Individuals belonging to at-risk work categories since they were vaccinated earlier than their age group (i.e., doctors, law-enforcement officers, and teachers).

Countries of origin were grouped according to the World Health Organization (WHO) regions classification based on regional distribution and the World Bank (WB) country-level economic classification by the income group based on the annual Atlas estimates of gross national income per capita (21). For the study analyses, the most recent WB economic classification (2022) was used (22).

For the purposes of this study, according to the United Nations (UN) definitions, individuals who live in Verona but who were not born in Italy were considered migrant people, regardless of their legal status, as the migration background was not available because the analyses examined only administrative data (23).

### 2.6. Endpoints

The primary study endpoint was the time-to-vaccination measure, which represented the time elapsed from the start of the vaccination campaign for each individual's risk category to the actual administration of the first dose of the vaccine. The secondary endpoints were the type of health facility used for vaccination and the distance traveled from the place of residence to the vaccination point used.

### 2.7. Statistical analysis

The descriptive analysis of the sample used frequencies and proportions for qualitative variables, mean and standard deviation, or median and quartile for quantitative variables. The sample distribution was tested with the *t*-test for continuous variables and with the chi-square (χ^2^) for categorical variables.

The first dependent variable, time-to-vaccination, was calculated as the difference between the actual date an individual received the first dose of COVID-19 vaccine and the date in which the local health authorities made vaccination reservations available for the corresponding age group.

The second dependent variable, distance (in kilometers) to the vaccination point used, was estimated with Q-GIS software through the network analysis algorithm provided by the “shortest path” (point-to-point) tool. The algorithm was selected to minimize the travel time, via the region's road network map, from the individual's municipality of residence to the vaccination point where the individual received the COVID-19 vaccine (24).

To explore the association between (1) time-to-vaccination and (2) distance between municipality of residence and vaccination point, and independent variables such as age, sex, and birth country, two multivariable linear regression models were constructed, one for each dependent variable. Average marginal effects (AME) and 0.95 confidence intervals were computed and presented as measures of potential impact.

Data were analyzed with the R software (version 4.1.2) and the QGIS software (version 3.16). A *p* < 0.05 was considered to be statistically significant.

## 3. Results

### 3.1. Sample characteristics

During the study period, 7,54,004 first doses were administered to individuals living in the province of Verona, of which 7,04,029 persons were 18 years and older. Considering an estimated adult population of 7,76,469, the vaccination rate with at least one dose of the COVID-19 vaccine was 90.7% at the end of 2021.

**Figure 1:**
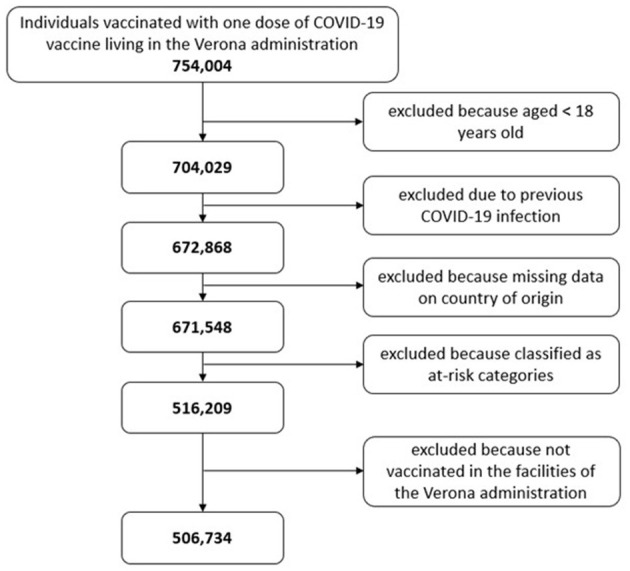
Flowchart of the included population and number of excluded individuals by exclusion criteria.

After applying the exclusion criteria, the sample population was 5,06,734 (Figure 1). The mean age of the whole sample was 51.2 years (SD19.4), with females making up nearly half of the sample at 2,46,399 (48.6%).

Migrants vaccinated in the province of Verona included in the analysis were 85,989 (17.0%). The majority came from the European Region (EUR, 42.3%), followed by the Eastern Mediterranean Region (EMR, 19.3%) and the South-East Asian Region (SEAR, 14.4%). Among migrants from EUR, more than half (66.7%) were from the following three countries: Romania (35.4%), Moldova (16.5%), and Albania (14.8%). The most represented countries by the WHO area are shown in Table 1.

**Table 1:** Distribution by the country of migrant people with at least one dose of COVID-19 vaccine included in the study and stratified by the WHO area.

**AFR (8,568)**	**N (%)**	**AMR (8,444)**	***N* (%)**
NIGERIA	2,854 (33.3%)	BRASIL	3,743 (44.3%)
GHANA	1,952 (22.8%)	ARGENTINA	800 (9.5%)
SENEGAL	1,352 (15.8%)	DOMINICAN REPUBLIC	702 (8.3%)
IVORY COAST	440 (5.1%)	COLOMBIA	643 (7.6%)
GUINEA	294 (3.4%)	PERU	602 (7.1%)
ALGERIA	265 (3.1%)	CUBA	541 (6.4%)
GAMBIA	240 (2.8%)	VENEZUELA	370 (4.4%)
MALI	233 (2.7%)	UNITED STATES OF AMERICA	210 (2.5%)
ETHIOPIA	170 (2.0%)	PARAGUAY	199 (2.4%)
TOGO	96 (1.1%)	ECUADOR	138 (1.6%)
CAMEROON	87 (1.0%)	MEXICO	129 (1.5%)
**SEAR (12,368)**	**N (%)**	**EMR (16,628)**	**N (%)**
SRI LANKA	6,042 (48.9%)	MOROCCO	12,530 (75.4%)
INDIA	5,393 (43.6%)	PAKISTAN	1,892 (11.4%)
BANGLADESH	697 (5.6%)	TUNISIA	1,194 (7.2%)
THAILAND	189 (1.5%)	EGYPT	278 (1.7%)
		LIBYA	265 (1.6%)
**EUR (36,363)**	**N (%)**	**WPR (3,618)**	**N (%)**
ROMANIA	12,861 (35.4%)	CHINA	2,780 (76.8%)
MOLDOVA	6,008 (16.5%)	PHILIPPINES	478 (13.2%)
ALBANIA	5,388 (14.8%)	VIETNAM	141 (3.9%)
GERMANY	1,312 (3.6%)	AUSTRALIA	92 (2.5%)
UKRAINE	1,199 (3.3%)	JAPAN	49 (1.4%)
SWITZERLAND	1,100 (3.0%)		
BOSNIA AND HERZEGOVINA	932 (2.6%)		
SERBIA	929 (2.6%)		
FRANCE	892 (2.5%)		
POLAND	824 (2.3%)		
RUSSIA	663 (1.8%)		
CROATIA	408 (1.1%)		
MACEDONIA	388 (1.1%)		
UNITED KINGDOM	384 (1.1%)		

AFR, African Region; AMR, Region of the Americas; SEAR, South-East Asian Region; EUR, European Region; EMR, Eastern Mediterranean Region; WPR, Western Pacific Region.

The mean age of the migrant population was 42.4 years (SD 13.3), ranging from 39.7 years (SD 11.4) for those from SEAR to 44.1 years (SD 13.9) for those from EUR. Females were generally underrepresented in the African Region (AFR, 32.7%) and the EMR (31.6%).

Based on the WB country-level economic classification, most migrants came from upper-middle (UMIC, 45.7%) and lower-middle (LMIC, 43.8%) income countries. Migrants from low-income countries (LIC) accounted for 1.8% of the whole migrant population. The mean age was 37.9 years (SD 16.2), 40.7 years (SD 12.3), and 42.7 years (SD 12.8) for people from LIC, LMIC, and UMIC, respectively, being significantly younger compared to migrants from HICs (mean age 52.3 years, SD 15.9, *p* < 0.001) and to Italy's general population (mean age 52.9 years, SD 20.0, *p* < 0.001).

Among these migrant groups, females were generally underrepresented in the LIC (23.0%) and LMIC (36.2%) groups.

### 3.2. Time-to-vaccination

**Figure 2:**
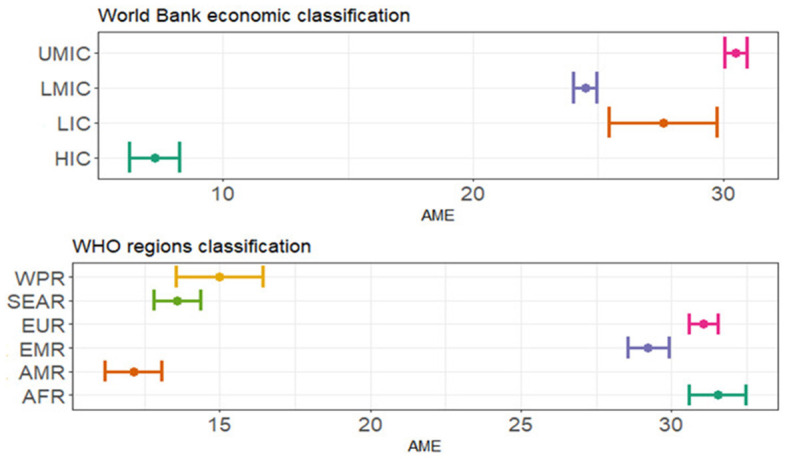
Graphic representation of average marginal effect (AME) with 0.95 confidential interval from multivariable linear regression with time-to-vaccination (in days) as a dependent variable and the country of birth, sex, and age as independent variables. HIC, high-income countries; UMIC, upper-middle income countries; LMIC, lower-middle income countries; LIC, low-income countries. AFR, African Region; AMR, Region of the Americas; SEAR, South-East Asian Region; EUR, European Region; EMR, Eastern Mediterranean Region; WPR, Western Pacific Region.

The mean time-to-vaccination for the whole sample was 46.9 days (SD 45.9, Table 2). In the Italian general population, the mean time-to-vaccination was 41.8 days (SD 43.5), while for the migrant group, it was 71.6 days (SD 49.1). Compared to the Italian general population, the time-to-vaccination was significantly higher for migrants (*p* < 0.001, Table 3). The AME of the time-to-vaccination compared to the Italian general population was higher by 27.6 days [0.95 CI 25.4–29.8], 24.5 days [0.95 CI 24.0–24.9], 30.5 days [0.95 CI 30.1–31.0], and 7.3 days [0.95CI 6.2-8.3] for migrants from LIC, LMIC, UMIC, and HIC, respectively (Figure 2, Table 3). Based on the WHO regions classification, the AME of the time-to-vaccination compared to the Italian group was higher by 31.5 days [0.95 CI 30.6–32.5], 31.1 days [0.95 CI 30.6–31.5], and 29.2 days [0.95 CI 28.5–29.9] for migrants from AFR, EUR, and EMR, respectively. By comparison, the AME for those from the Western Pacific Region (WPR, 14.9 days [0.95 CI 13.5–16.4]), the SEAR (13.6 days [0.95 CI 12.8–14.4]), and the Region of the Americas (AMR, 12.1 days [0.95 CI 11.2–13.1]) were lower. Overall, after adjusting for sex and birth country, as age increased, time-to-vaccination decreased yielding an AME of 0.38 days [0.95 CI 0.37–0.39] (*p* < 0.001, Table 3).

**Table 2:** Descriptive characteristics of the individuals with at least one dose of COVID-19 vaccine included in the study stratified by the World Bank income group and the WHO area.

	**Italy (*n* = 420,747)**	**HIC (*n* = 7,416)**	**UMIC (*n* = 39,303)**	**LMIC (*n* = 37,687)**	**LIC (*n* = 1,581)**	**Overall (*n* = 506,734)**
**Sex**						
Female	206,122 (49.0%)	4,487 (60.5%)	21,796 (55.5%)	13,631 (36.2%)	363 (23.0%)	246,399 (48.6%)
Male	214,625 (51.0%)	2,929 (39.5%)	17,507 (44.5%)	24,056 (63.8%)	1,218 (77.0%)	260,335 (51.4%)
**Age (years)**						
Mean (SD)	53.0 (20.0)	52.3 (15.9)	42.3 (12.8)	40.7 (12.3)	38.0 (16.2)	51.2 (19.4)
**Vaccination point**						
Hub	391,293 (93.0%)	6,902 (93.1%)	36,340 (92.5%)	35,334 (93.8%)	1,383 (87.5%)	471,252 (93.0%)
Family doctor	17,581 (4.2%)	301 (4.1%)	992 (2.5%)	429 (1.1%)	17 (1.1%)	19,320 (3.8%)
Pharmacy	5,416 (1.3%)	120 (1.6%)	1,248 (3.2%)	1,016 (2.7%)	73 (4.6%)	7,873 (1.6%)
LHUs	3,529 (0.8%)	42 (0.6%)	521 (1.3%)	646 (1.7%)	85 (5.4%)	4,823 (1.0%)
Nursing home	2,801 (0.7%)	42 (0.6%)	163 (0.4%)	174 (0.5%)	19 (1.2%)	3,199 (0.6%)
Law-enforcement facilities	127 (0.0%)	9 (0.1%)	39 (0.1%)	88 (0.2%)	4 (0.3%)	267 (0.1%)
**Vaccine type**						
COMIRNATY	320,092 (76.1%)	5,526 (74.5%)	29,424 (74.9%)	29,395 (78.0%)	1,179 (74.6%)	385,616 (76.1%)
MODERNA	57,403 (13.6%)	1,088 (14.7%)	8,499 (21.6%)	7,053 (18.7%)	364 (23.0%)	74,407 (14.7%)
ASTRAZENECA	31,085 (7.4%)	543 (7.3%)	752 (1.9%)	630 (1.7%)	15 (0.9%)	33,025 (6.5%)
JANSSEN	12,167 (2.9%)	259 (3.5%)	628 (1.6%)	609 (1.6%)	23 (1.5%)	13,686 (2.7%)
**Time-to-vaccination (days)**						
Mean (SD)	41.8 (43.5)	49.2 (50.3)	76.3 (53.2)	71.0 (43.0)	75.2 (41.8)	46.9 (45.9)
**Distance traveled (Km)**						
Mean (SD)	13.9 (12.1)	15.4 (14.1)	13.8 (12.8)	13.5 (12.4)	12.6 (11.9)	13.8 (12.2)
	**EUR (*****n** =* **36,362)**	**EMR (*****n** =* **16,628)**	**SEAR (*****n** =* **12,368)**	**AFR (*****n** =* **8,567)**	**AMR (*****n** =* **8,444)**	**WPR (*****n** =* **3,618)**
**Sex**						
Female	20,503 (56.4%)	5,260 (31.6%)	4,717 (38.1%)	2,801 (32.7%)	5,081 (60.2%)	1,915 (52.9%)
Male	15,859 (43.6%)	11,368 (68.4%)	7,651 (61.9%)	5,766 (67.3%)	3,363 (39.8%)	1,703 (47.1%)
**Age (years)**						
Mean (SD)	44.1 (13.9)	41.1 (13.0)	39.7 (11.4)	40.5 (12.8)	42.5 (13.7)	43.8 (12.1)
**Vaccination point**						
Hub	33,647 (92.5%)	15,643 (94.1%)	11,741 (94.9%)	7,731 (90.2%)	8,006 (94.8%)	3,191 (88.2%)
Pharmacy	1,124 (3.1%)	360 (2.2%)	328 (2.7%)	338 (3.9%)	224 (2.7%)	83 (2.3%)
Family doctor	1,190 (3.3%)	215 (1.3%)	101 (0.8%)	97 (1.1%)	109 (1.3%)	27 (0.7%)
LHUs	216 (0.6%)	290 (1.7%)	109 (0.9%)	336 (3.9%)	55 (0.7%)	288 (8.0%)
Nursing home	144 (0.4%)	49 (0.3%)	88 (0.7%)	44 (0.5%)	44 (0.5%)	29 (0.8%)
Law-enforcement facilities	41 (0.1%)	71 (0.4%)	1 (0.0%)	21 (0.2%)	6 (0.1%)	0 (0.0%)
**Vaccine type**						
COMIRNATY	26,774 (73.6%)	13,259 (79.7%)	9,762 (78.9%)	6,381 (74.5%)	6,457 (76.5%)	2,891 (79.9%)
MODERNA	7,791 (21.4%)	2,731 (16.4%)	2,344 (19.0%)	1,935 (22.6%)	1,595 (18.9%)	608 (16.8%)
ASTRAZENECA	1,067 (2.9%)	335 (2.0%)	119 (1.0%)	129 (1.5%)	228 (2.7%)	62 (1.7%)
JANSSEN	730 (2.0%)	303 (1.8%)	143 (1.2%)	122 (1.4%)	164 (1.9%)	57 (1.6%)
**Time-to-vaccination (days)**						
Mean (SD)	76.2 (55.7)	75.7 (45.2)	60.6 (37.5)	78.2 (40.8)	57.9 (45.6)	60.3 (41.4)
**Distance traveled (Km)**						
Mean (SD)	14.2 (12.8)	15.2 (12.6)	12.3 (12.3)	12.5 (12.1)	14.2 (13.9)	11.2 (12.2)

HIC, high-income countries; UMIC, upper-middle income countries; LMIC, lower-middle income countries; LIC, low-income countries. AFR, African Region; AMR, Region of the Americas; SEAR, South-East Asian Region; EUR, European Region; EMR, Eastern Mediterranean Region; WPR, Western Pacific Region; SD, standard deviation.

**Table 3:** Results of the two linear regression models fitted on time-to-vaccination (days) as dependent variable and birth country, age and sex as potential determinants.

	**Estimate**	**Std. error**	***t* value**	***p*-value**
**WB classification**				
(Intercept)	61.2	0.2	305.1	< 0.001
HIC	7.3	0.5	14.1	< 0.001
LIC	27.6	1.1	25.0	< 0.001
LMIC	24.5	0.2	102.3	< 0.001
UMIC	30.5	0.2	130.3	< 0.001
Age	−0.4	0.0	−114.1	< 0.001
Sex (male)	0.7	0.1	5.4	< 0.001
**WHO classification**				
(Intercept)	61.8	0.2	308.5	< 0.001
AFR	31.5	0.5	65.7	< 0.001
AMR	12.1	0.5	25.1	< 0.001
EMR	29.2	0.3	83.8	< 0.001
EUR	31.1	0.2	128.7	< 0.001
SEAR	13.6	0.4	33.8	< 0.001
WPR	15.0	0.7	20.5	< 0.001
Age	−0.4	0.0	−117.4	< 0.001
Sex (male)	0.6	0.1	4.9	< 0.001

The birth country was grouped according to the World Bank (WB) country-level economic and to the World Health Organization (WHO) regions classifications. Reference level in both models was “Italy”.

HIC, high-income countries; UMIC, upper-middle income countries; LMIC, lower-middle income countries; LIC, low-income countries. AFR, African Region; AMR, Region of the Americas; SEAR, South-East Asian Region; EUR, European Region; EMR, Eastern Mediterranean Region; WPR, Western Pacific Region; SD, standard deviation.

### 3.3. Access to vaccination points

**Figure 3:**
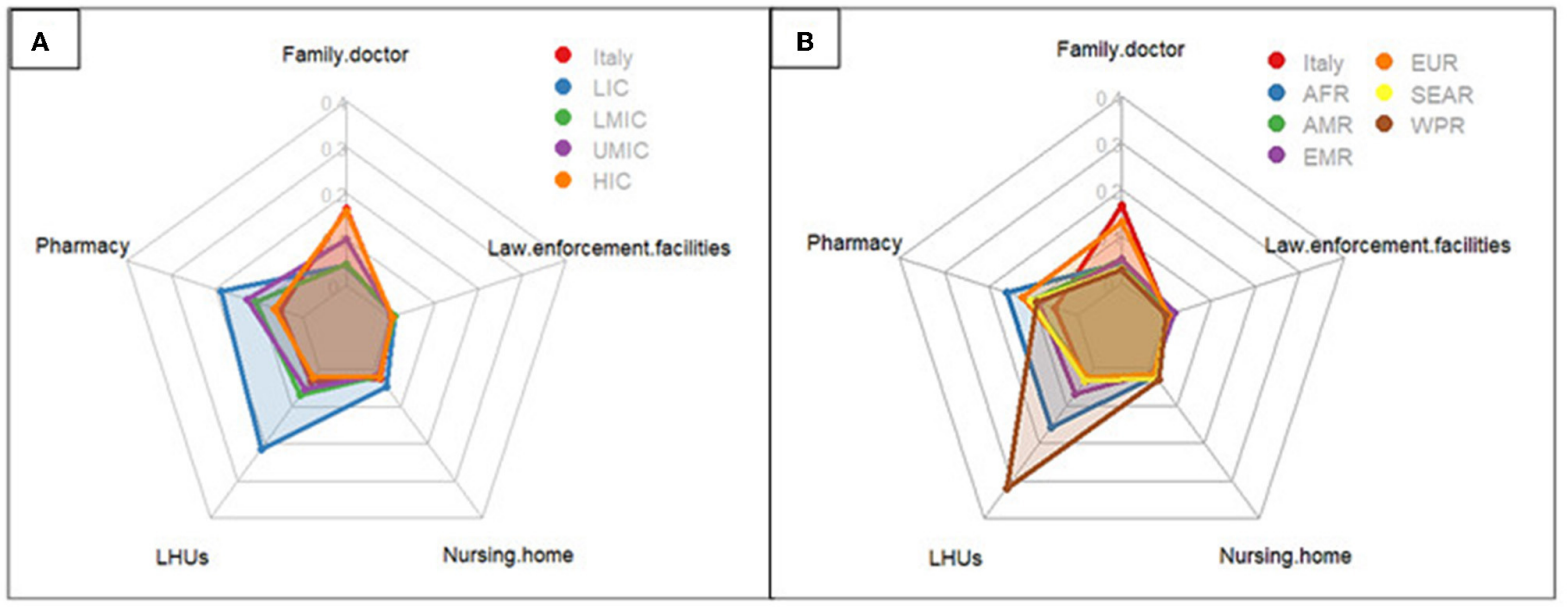
Radar plot of the percentage of vaccination points, other than hub centers, used by individuals distinguished by the country of origin classified based on the World Bank income group **(A)** and WHO area **(B)**. HIC, high-income countries; UMIC, upper-middle income countries; LMIC, lower-middle income countries; LIC, low-income countries. AFR, African Region; AMR, Region of the Americas; SEAR, South-East Asian Region; EUR, European Region; EMR, Eastern Mediterranean Region; WPR, Western Pacific Region; LHUs, Local Health Units.

The most frequently used vaccination points among both the Italians and migrant populations were the Hub centers located across the province (Table 2). For vaccination points other than Hubs centers, the LHUs were used as an alternative site by migrants from the WPR (8.0%), the AFR (3.9%), and the EMR (1.7%). Migrants also tended to use pharmacies more compared to the Italian general population (Figure 3, Table 2). Among migrants from EUR and the Italian general population, family doctors were also a frequently used alternative (4.2% and 3.3%, respectively). Based on the WB country-level economic classification, access to vaccination points was similar among Italians and migrants from HIC, while those from other economic groupings used more often pharmacies (3.2% UMIC, 2.7% LMIC, 4.6% LIC) and LHUs (1.3% UMIC, 1.7% LMIC, 5.4% LIC).

The mean distance traveled to reach a vaccination point was 13.7 km (SD 12.2) in the overall sample (Table 2). The distance was shorter for migrants from LIC and LMIC, yielding an AME of 2.9 km [2.2–3.6] and 1.5 km [1.4–1.7], respectively, as compared to the Italian population; the mean distance traveled to reach a vaccination point was greater in migrants from HIC (AME: 1.5 km [0.95 CI 1.2–1.9], p < 0.001, Table 4) compared to the Italian population. Based on the WHO regions classification, migrants from the WPR, the SEAR, and the AFR traveled a shorter distance to a vaccination point than Italians yielding an AME of 3.3 km [0.95 CI 2.9–3.8], 2.7 km [0.95 CI 2.6–3.0], and 2.7 km [0.95 CI 2.4–3.0], respectively (p < 0.001, Table 4).

**Table 4:** Results of the two linear regression models fitted on the distance between the residence address and the vaccination point used as dependent variable, and birth country, age and sex as potential determinants.

	**Estimate**	**Std. Error**	***t* value**	***p*-value**
**WB classification**				
(Intercept)	17.7	0.1	260.2	< 0.001
HIC	1.5	0.2	8.7	< 0.001
LIC	−2.9	0.4	−8.1	< 0.001
LMIC	−1.5	0.1	−19.4	< 0.001
UMIC	−1.0	0.1	−13.4	< 0.001
Age	−0.1	0.0	−73.7	< 0.001
Sex (male)	0.7	0.0	17.5	< 0.001
**WHO area classification**				
(Intercept)	17.6	0.1	260.0	< 0.001
AFR	−2.7	0.2	−17.3	< 0.001
AMR	−0.5	0.2	−2.9	0.004
EMR	0.2	0.1	1.8	0.07
EUR	−0.5	0.1	−6.6	< 0.001
SEAR	−2.7	0.1	−21.1	< 0.001
WPR	−3.3	0.2	−14.4	< 0.001
Age	−0.1	0.0	−73.2	< 0.001
Sex (male)	0.7	0.0	17.2	< 0.001

The birth country was grouped according to the World Bank (WB) country-level economic and to the World Health Organization (WHO) regions classifications. Reference level in both models was “Italy”.

HIC, high-income countries; UMIC, upper-middle income countries; LMIC, lower-middle income countries; LIC, low-income countries. AFR, African Region; AMR, Region of the Americas; SEAR, South-East Asian Region; EUR, European Region; EMR, Eastern Mediterranean Region; WPR, Western Pacific Region; SD, standard deviation.

## 4. Discussion

The present study explored access to the COVID-19 vaccine during the first year of the vaccination campaign in the province of Verona. Although in Italy, the public health system has guaranteed free access to COVID-19 vaccination for the entire population regardless of legal status, significant differences in vaccine access based on migration background were found, as already emerged in other European and non-European countries (25, 26). Compared to the Italian general population, a longer time-to-vaccination was found for migrants living in Verona, with significant discrepancies by birth country (WHO regions classifications) and by country-level economic categories (WB economic classification). Moreover, the time-to-vaccination also decreased as age increased, after adjusting for sex and birth country in the analytic models.

At the end of 2021 in Italy, vaccination rate with at least one dose of the COVID-19 vaccine ranged from 85.4% in people aged 40–49 years old to 95.8% in over 80 years old; for the study sample, the vaccination rate was 90.7% (27). Although some studies showed that vaccination rate was reasonably high and similar between the general population and migrants, our results suggest that this goal was achieved at different times and with different types of vaccination points used by the two groups (28). In fact, the time-to-vaccination for migrants was longer by 71%, i.e., by almost 30 days (41.8 vs. 71.6), suggesting that migrants were exposed more frequently to the SARS-CoV-2 infection, without the opportunity to obtain even partial immunization, on average 1 month longer compared to the general population.

Previous research has shown that the birth country of migrant groups is a reliable and key factor to account not only for monitoring health outcomes (morbidity and mortality) but also for understanding health attitudes and behavior (29). To explore birth country's influence on COVID-19 vaccine access, this study stratified the study sample using the WHO regions and the WB country-level economic classifications. The first classification highlights the potential influence of different cultural factors and behaviors that may impact vaccine receptivity and uptake, while the second classification provides an analysis of the potential impact of the pre-migration socio-economic characteristics that may have influenced healthcare and other services access after resettlement (30).

Differences were found in the distance traveled to get to the vaccination point used: migrants from LIC and LMIC traveled shorter distances than Italians, while those from HIC traveled higher distances. Similarly, based on the WHO regions classification, people from the AFR, the SEAR, and the WPR traveled fewer kilometers than Italians. As a result, the distance to healthcare facilities was a factor in access to vaccination, and the birth country had an influence on it. Additionally, vaccination points also differed by the type of facility used. Excluding the larger hub centers, the most frequently used vaccination points for all groups considered Italians and migrants from the EUR and from HIC who were vaccinated in higher percentages at family doctors than the other groups and in lower percentages at pharmacies and LHU units. Hub centers were typically located in large pre-existing facilities, such as gyms and fair centers, that could usually support high work volumes and had large car parks but far from urban centers. This also accounts for the very long mean traveled distance of 13.8 km found in the overall sample. By contrast, LHUs and pharmacies, which subsequently started to provide the vaccination, were more widely distributed throughout the local communities. In this study, migrants often chose or were more likely to use venues closer to their homes as they could not travel greater distances. This finding is consistent with other research on healthcare access: travel distance, lack of flexibility at work, childcare responsibilities, limited transport options, more difficult transportation, and indirect costs all present physical and time barriers to obtaining health services such as vaccination (31). Consequently, the Verona experience with the COVID-19 vaccination campaigns provided lessons learned, suggesting that offering alternative site options for vaccination, including pharmacies, local health units, and primary care doctors, as well as greater investment in public transports may be useful strategies for improving vaccine access and uptake for vulnerable populations during a global public health emergency.

Different factors may have also contributed to delays in receiving a vaccine. For example, the health system itself may be viewed with distrust and fear among migrant populations. Although the COVID-19 vaccine was a guaranteed medical service, it could not be provided without the persons being entered into the health register, making it difficult to ensure that certain categories, i.e., undocumented migrants, received adequate care (32).

To access the vaccination, a reservation was required. The reservation could only be made online, only if in possession of an identification number, on a site with no web app and written only in Italian. A digital gap, i.e., a lower digital competence previously observed in migrants, reduced access to digital technologies, lower literacy, and the existence of language limitations may have contributed to the delay or decrease in vaccination adherence (33). Equitable access to care could therefore be achieved also through public health authorities' commitment to build easy and effective pathways for the entire population to obtain it, such as multilingual websites. Implementing healthcare accessibility for migrants could improve their health outcomes, anticipate patient access and continuity of care, and additionally reduce costs (34).

The vaccine uptake is also influenced by vaccination hesitancy, an element that has individual and cultural roots and is more prevalent in certain populations, such as those in Eastern Europe (35). Indeed, in our sample, migrants from EUR, mainly including East Europe countries, had higher time-to-vaccination compared to the Italian general population. All these factors interconnect with the concept of health literacy, which is influenced by culture and society, the education system, and the healthcare system (36). Previous research has shown lower health literacy among ethnic minorities or migrants (37). Health literacy, however, is also influenced by the way health information is communicated to people when they try to determine what they need to do to take care of their health. Therefore, it is necessary to invest in improving health literacy so that efforts to improve quality, reduce costs, and reduce inequities can be successful (36).

The last two elements of vaccine access and uptake that were examined by this study were the contribution of sex and age. In the study sample, women outnumber men in HICs and UMIC migrants. Female migration to Europe has increased significantly (the so-called “feminization of migration”). This could be explained by the growth of highly skilled migrants, the demand for conventionally gender-selective jobs (e.g., healthcare) and domestic work, or human trafficking (38). With regard to the correlation between time-to-vaccination and age, it was observed that as age increased, for each of the WHO regions and county-level economic groups, this period decreased. The higher perceived risk of COVID-19 infection in the older adults appears to be a factor leading to earlier access to treatment than in younger age groups (39). The high vaccination rate found in this study may also have been influenced by the introduction of the “Green pass” (a certificate of having received the COVID-19 vaccine or having recovered from the disease), which was progressively made mandatory not only to access healthcare facilities but also for recreational activities (access to restaurants, museums, gyms, etc.). The requirement for this certificate has been later extended to all public and private workers, as well as to all people aged over 50 years, making it difficult for an individual to work and thus secure an economic income for themselves and their family without having been vaccinated (40).

This study has several limitations. First, it was a retrospective study focusing on a single province, so the results cannot be generalized to the entire Italian national context. Second, it was assumed that latent factors influencing access (e.g., organizational difficulties in the regional health system related to the extraordinary nature of the vaccination campaign) were common across all country groups considered. Moreover, the birth country (based on both the WHO regions and the WB country-level economic classifications) was considered as a factor affecting access to healthcare services. Although this relationship is known from the literature, it has limitations related to the interpersonal variables that depend on several factors, such as post-migration integration, educational level, and household income (29). In addition, information regarding the legal status or citizenship of the people involved was not available in this study. This prevented a more extensive analysis of the barriers to vaccination for the migrant population and could have biased some estimations. Last, the analysis was based exclusively on data recorded in the Regional Vaccination Information System; therefore, both the quantity and quality of the information in the register itself may be affected by errors in data entry or may not be readily updated. However, these limitations are intrinsic to a retrospective analysis of claims databases.

## 5. Conclusion

This study showed that the migrant population in the province of Verona received COVID-19 vaccination later than the Italians and those from LIC used different vaccination points, traveling shorter distances to reach them. These two findings suggest that offering health services free of charge is not sufficient to ensure that the entire population has access to them, and this disparity particularly affects groups already considered at risk, such as migrants. In the local setting covered by this study, investing in community health and social care could increase outreach vaccination programs for the most fragile populations. At the same time, promoting a linguistically inclusive healthcare system (i.e., booking software in different languages) and training healthcare workers to improve their cultural competence could promote vaccination adherence in different ethnic groups. Similarly, tailored vaccination communication for the migrant population should be promoted by health authorities and the media. Equity in access to COVID-19 vaccines, as called for by the WHO and the European Center for Disease Prevention and Control, does not only comprise the achievement of equal vaccination rate but also that all people have the same opportunity to access it in due time (41). This should be taken into account by public health stakeholders, as a delay in a health outcome for one part of the society, such as exposure to infection for migrants, could affect the overall course of the vaccination campaign and the epidemic.

## Data availability statement

The datasets generated and/or analyzed during the current study are available from the corresponding author upon reasonable request. Requests to access these datasets should be directed to roberto.benoni90@gmail.com.

## Ethics statement

The studies involving human participants were reviewed and approved by Ethical Committee of Azienda ULSS n. 9 Scaligera. Written informed consent for participation was not required for this study in accordance with the national legislation and the institutional requirements.

## Author contributions

RB and AS conceptualized and designed the study and made substantial contributions to original writing. RB was responsible for data analysis. FMo contributed to the interpretation of the data and the original writing. FMa and CP contributed to data collection and interpretation. LA contributed to data collection. VC and ST reviewed the study critically and contributed to data interpretation. All authors contributed to the article and approved the submitted version.
